# A Memristive Hyperchaotic System without Equilibrium

**DOI:** 10.1155/2014/368986

**Published:** 2014-07-15

**Authors:** Viet-Thanh Pham, Christos Volos, Lucia Valentina Gambuzza

**Affiliations:** ^1^School of Electronics and Telecommunications, Hanoi University of Science and Technology, 01 Dai Co Viet, Hanoi, Vietnam; ^2^Physics Department, Aristotle University of Thessaloniki, 54124 Thessaloniki, Greece; ^3^University of Catania, Viale A. Doria 6, 95125 Catania, Italy

## Abstract

A new memristive system is presented in this paper. The peculiarity of the model is that it does not display any equilibria and exhibits periodic, chaotic, and also hyperchaotic dynamics in a particular range of the parameters space. The behavior of the proposed system is investigated through numerical simulations, such as phase portraits, Lyapunov exponents, and Poincaré sections, and circuital implementation confirmed the hyperchaotic dynamic.

## 1. Introduction

Since the first hyperchaotic attractor introduced by Rössler [1], hyperchaotic systems have been intensively studied due to their potential applications in many fields of interest. Usually a hyperchaotic circuit is a four-dimensional system, it is characterized by more than one positive Lyapunov exponent and, thus, presents a higher level of complexity with respect to chaotic system allowing to use it in diverse applications such as cryptosystems [2], neural networks [3], secure communications [4, 5], or laser design [6].

After the realization of a solid-state thin film two-terminal memristor at Hewlett-Packard Labs [7], a considerable number of potential memristor-based applications have been reported like high-speed low-power processors [8], adaptive filter [9], pattern recognition systems [10], associative memory [11], neural networks [12, 13], programmable analog integrated circuits [14], and so on [15, 16]. Interestingly, the intrinsic nonlinear characteristic of memristor has been exploited in implementing novel chaotic oscillators with complex dynamics [17, 18]. It is very interesting to ask naturally whether there exists a memristor-based system that is hyperchaotic. Some authors have recently answered this question, Buscarino et al. [19] designed a hyperchaotic oscillator by extending the HP memristor-based canonical Chua's oscillator [20]. However, from the view of mathematical simplicity, this oscillator is complicated since it is a six-dimensional circuit. In [21], starting from a memristor-based canonical Chua's circuit, a five-dimensional hyperchaotic oscillator was introduced, while a four-dimensional hyperchaotic memristive system with a line equilibrium was reported by Li et al. [22]. The last example belongs to a new category of chaotic systems with hidden attractors. According to a new classification of chaotic dynamics [23, 24], there are two types of attractors: self-excited attractors and hidden attractors. A self-excited attractor has a basin of attraction that is excited from unstable equilibria. In contrast, hidden attractor cannot be found by using a numerical method in which a trajectory started from a point on the unstable manifold in the neighbourhood of an unstable equilibrium [23]. Studying hyperchaotic systems with hidden attractors is still an open research direction [25, 26].

Motivated by complex dynamical behaviors of hyperchaotic systems, noticeable characteristics of memristor, and unknown features of hidden attractors, a novel memristor-based hyperchaotic system without equilibrium is proposed in this paper. The paper is organized as follows. In the next section, the model of memristive device is introduced. This memristive device is used as the main component in the new memristive system, which is proposed in Section 3.  Section 4 presents the circuital implementation of the new proposed memristive system. Finally, the conclusive remarks are drawn in the last section.

## 2. Model of Memristive Device

Chua and Kang [27] introduced memristive system by generalizing the original definition of a memristor. In general, a memristive system is described by

(1) w˙m=F(wm,um,t),ym=G(wm,um,t)um,

where *u*_*m*_, *y*_*m*_, and *w*_*m*_ denote the input, output, and state of the memristive system, respectively. The function *F* is a continuous *n*-dimensional vector function and *G* is a continuous scalar function. Based on the definition of memristive system, a memristive device is proposed by the following form:

(2) w˙m=um,ym=(1+0.24wm2−0.0016wm4)um.

Hence *G* is a fourth degree polynomial function. In order to investigate the fingerprints of memristive device (2), an external bipolar period signal is applied across its terminals. The external sinusoidal stimulus is given by

(3) $$\begin{array}{c} u_{m}=U\sin\left(2\pi ft\right), \end{array}$$

where *U* is the amplitude and *f* is the frequency. From the first equation of (2), the state variable of the memristive device is described by

**Figure 1:**
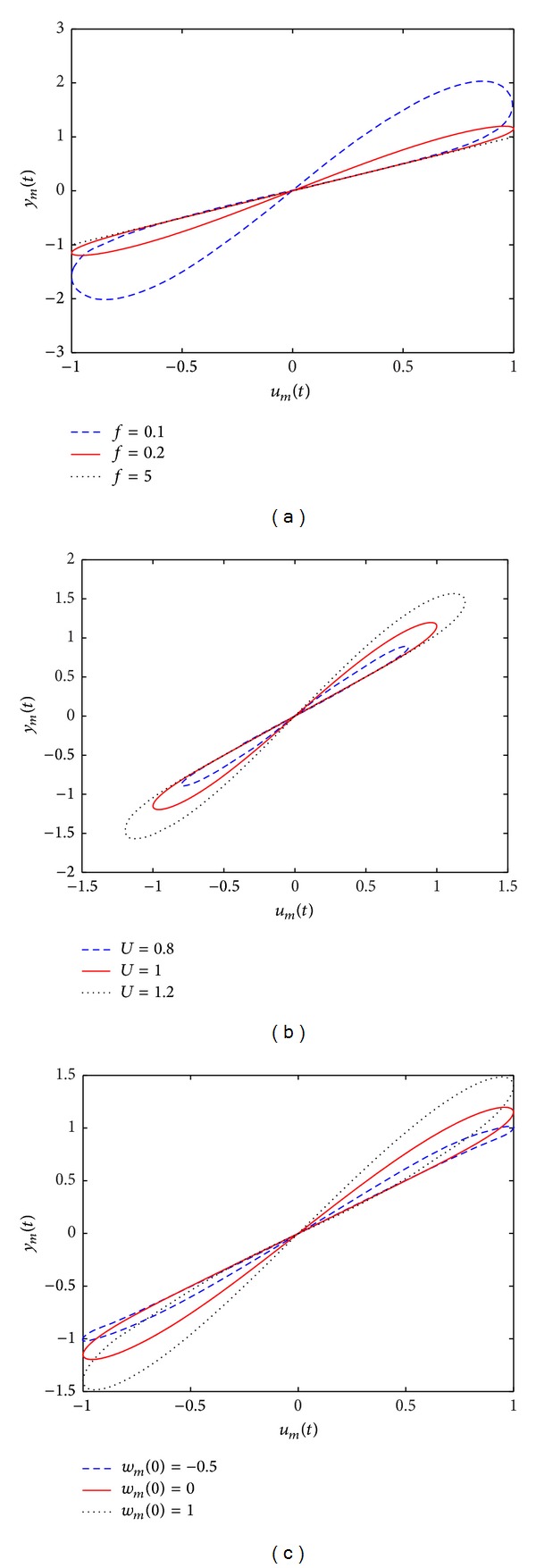
Hysteresis loops of the proposed memristive device (2) driven by a sinusoidal stimulus (3) when (a) *U* = 1, *w*_*m*_(0) = 0, and varying frequency *f*, (b) *f* = 0.2, *w*_*m*_(0) = 0, and changing input amplitude *U*, and (c) *U* = 1, *f* = 0.2, and using different initial states *w*_*m*_(0).

(4) wm(t)=∫−∞tum(τ)dτ=wm(0)+∫0tUsin⁡(2πfτ)dτ  =wm(0)+U2πf(1−cos⁡⁡(2πft)),

where *w*_*m*_(0) = ∫_−*∞*_^0^ *u*_*m*_(*τ*)*dτ* is the initial condition of the internal state variable *w*_*m*_. Substituting (3) and (4) into (2), it is easy to derive the output of the memristive device *y*_*m*_. Therefore, the output *y*_*m*_ depends on the frequency and amplitude of the applied input stimulus.  Figure 1(a) shows the hysteresis loop of the memristive device (2) when driven by a periodic signal (3) with different frequencies. Obviously, the proposed memristive device exhibits a “pinched hysteresis loop” in the input-output plane [28, 29]. In addition, when the excitation frequency increases, the hysteresis lobe area decreases monotonically. Moreover, when the frequency is adequately large, the pinched hysteresis loop shrinks to a single-valued function. It is worth noting that the hysteresis loop of the memristive device (2) pinched at different input amplitudes (see Figure 1(b)). Additionally, the output *y*_*m*_ also depends on the initial state of memristive device, as depicted in Figure 1(c). Thus, according to [30, 31] the three main fingerprints of memristive system have been observed in the proposed memristive device (2).

## 3. New Memristive Hyperchaotic System

Based on the introduced memristive device (2) in the previous section, a novel four-dimensional system is proposed as follows:

(5) x˙=−10x−aum−umz,u˙m=−6x+1.2xz+0.1ym−b,z˙=−z−1.2xum,w˙m=um,

where *a* and *b* are positive real parameters and *y*_*m*_ is the output of the memristive device as mentioned in (2). New system (5) is similar to the 4D memristive system in [22], but the function *G* of the memristive device is a fourth degree polynomial function.

When *b* = 0, system (5) has the line equilibrium E(0,0, 0, *w*_*m*_). It is worth noting that system (5) is hyperchaotic for different values of *a*. For example, when *a* = 5, *b* = 0, and the initial condition (*x*(0), *u*_*m*_(0), *z*(0), *w*_*m*_(0)) = (0,0.01,0.01,0), hyperchaos is obtained due to the fact that system (5) has more than one positive Lyapunov exponent *λ*_1_ = 0.1364, *λ*_2_ = 0.0071, *λ*_3_ = 0, and *λ*_4_ = −10.8584. In other word, system (5) becomes a hyperchaotic system with a line of equilibria based on a memristive device. As a result, this hyperchaotic system can be considered as a dynamical system with hidden attractor [24, 25] because it is impossible to verify the chaotic attractor by choosing an arbitrary initial condition in the vicinity of the unstable equilibria [23].

**Figure 2:**
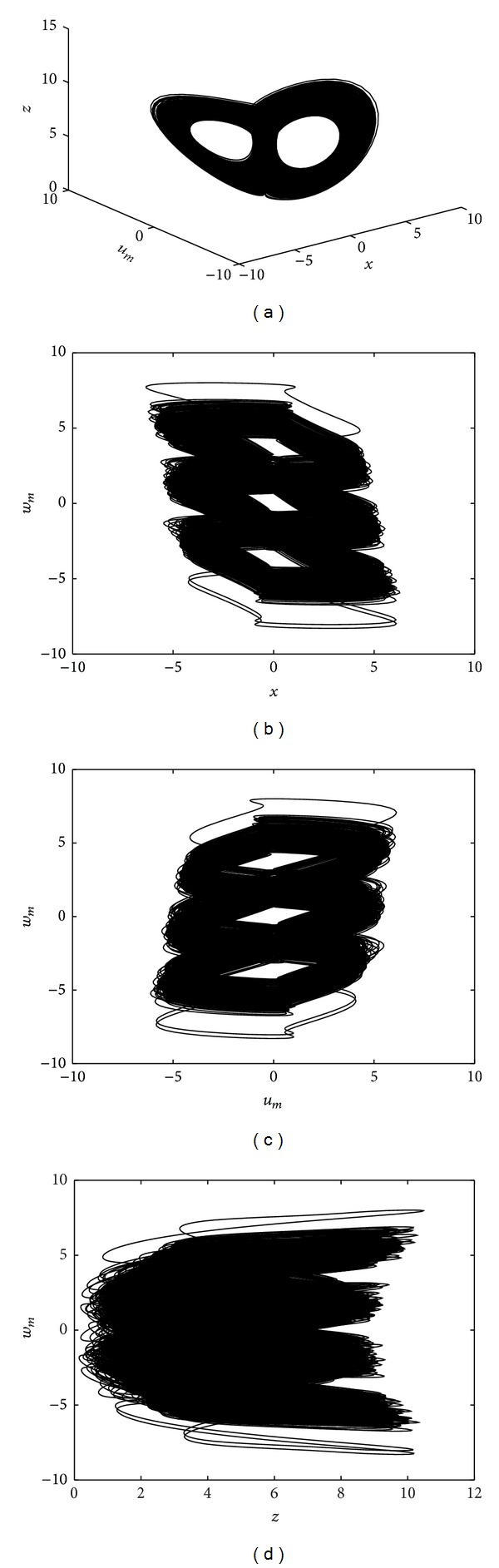
Hyperchaotic attractor without equilibrium obtained from system (5) for *a* = 5, and *b* = 0.001 (a) in the *x* − *u*_*m*_ − *z* space, (b) in the *x* − *w*_*m*_ plane, (c) in the *u*_*m*_ − *w*_*m*_ plane, and (d) in the *z* − *w*_*m*_ plane.

**Figure 3:**
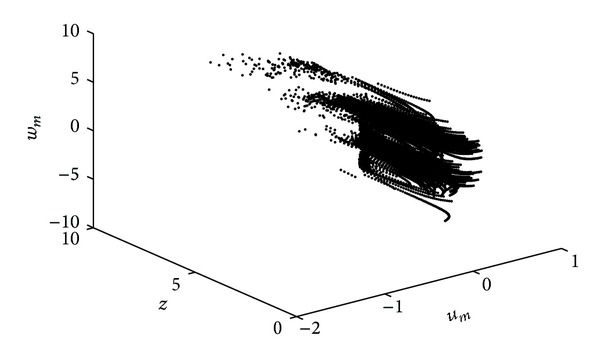
Poincaré map in the *u*_*m*_ − *z* − *w*_*m*_ space plane when *x* = 0 for *a* = 5 and *b* = 0.001.

When *b* ≠ 0, it can be noticed that the proposed system (5) possesses no equilibrium points. Interestingly, when *a* = 5, *b* = 0.001, and the initial condition (*x*(0), *u*_*m*_(0), *z*(0), *w*_*m*_(0)) = (0,0.01,0.01,0), the new system (5) can exhibit a hyperchaotic attractor without equilibrium (see Figure 2). As it can be seen from the Poincaré map in Figure 3, the memristive system (5) has a rich dynamical behavior. Here the Poincaré map is plotted in the 3D *u*_*m*_ − *z* − *w*_*m*_ space when *x* = 0.

**Figure 4:**
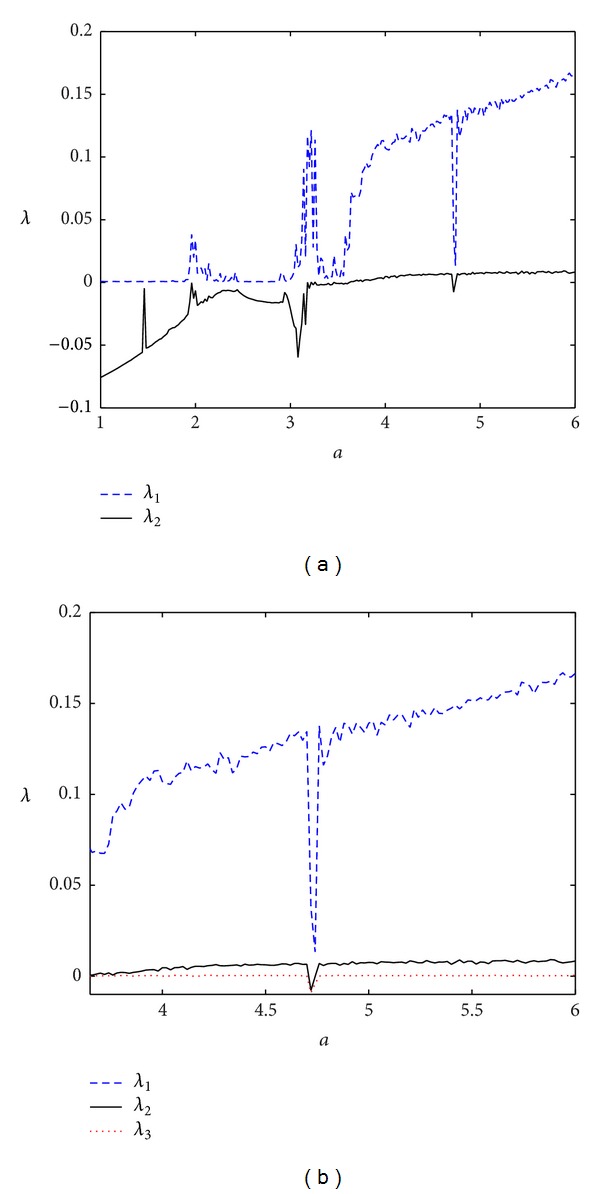
Lyapunov exponents of system (5) versus *a* for *b* = 0.001, (a) two largest Lyapunov exponents *λ*_1_, *λ*_2_; (b) three largest Lyapunov exponents *λ*_1_, *λ*_2_, and *λ*_3_.

**Figure 5:**
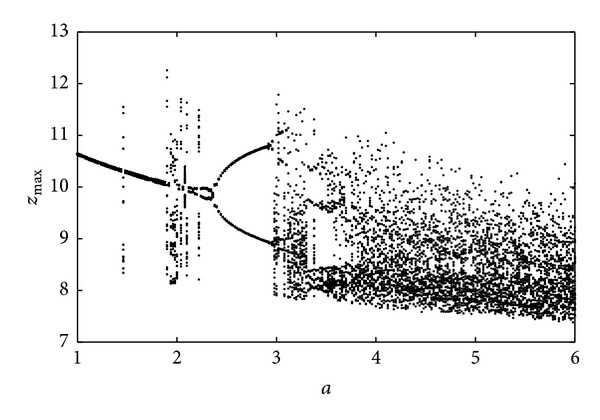
Bifurcation diagram of *z*_*max*_ with *b* = 0.001 and *a* as varying parameter.

It is well known that Lyapunov exponents measure the exponential rates of the divergence and convergence of nearby trajectories in the phase space of the chaotic system [32]. A hyperchaotic system is considered as a chaotic system with more than one positive Lyapunov exponent [1]. In order to have detailed view of the novel memristive system (5), Lyapunov exponents have been calculated using the algorithm in [33] and are predicted in Figure 4. Obviously, Lyapunov spectrum clearly indicates that there are some windows of limit cycles (*a* ∈ [1,1.92], (2.42,2.88]), of chaotic behavior (*a* ∈ (1.92,2.42], (2.88,3.68)) and of hyperchaotic behavior (*a* > 3.68). In addition, the corresponding bifurcation diagram, which is obtained by plotting the local maxima of the state variable *z*(*t*), is shown in Figure 5. The spectrum of Lyapunov exponents reported in Figure 4 well agrees with the bifurcation diagram of Figure 5.

## 4. Circuit Implementation of the Memristive Hyperchaotic System

Implementation of chaotic/hyperchaotic systems by using electronic circuits provides an effective approach for discovering dynamics of such system. This physical approach can avoid the uncertainties arising from systematic and statistical errors in numerical simulations [34]. Moreover, circuital realization of theoretical model plays an important role in practical chaos-based applications such as secure communications [35], random numbers generator [36], or path planning for autonomous robots [37].

**Figure 6:**
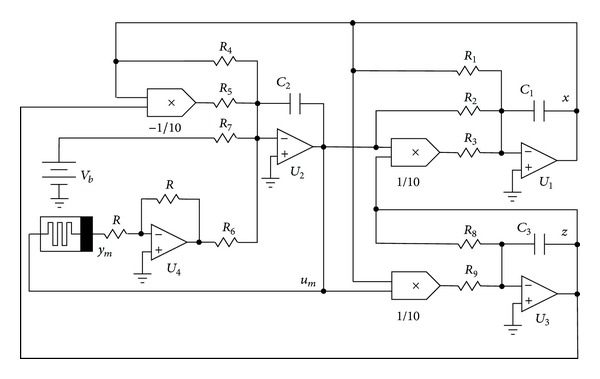
Circuital schematic of the new hyperchaotic system without equilibrium (5) based on the memristive device (2).

**Figure 7:**
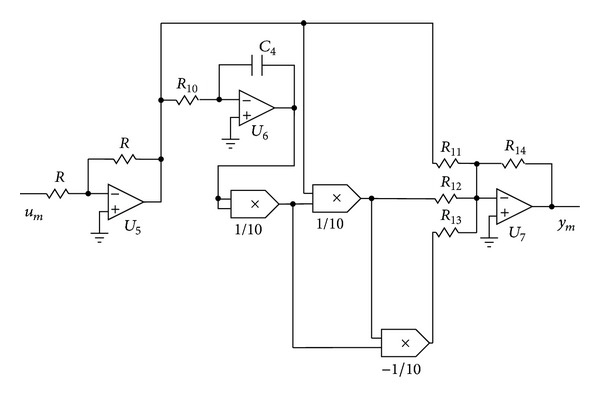
Circuitry realization which emulates the memristive device (2).

Therefore, in this section, a circuital realization of system (5) is reported to illustrate the feasibility and correctness of the theoretical model. The designed circuit is shown in Figure 6 where the variables *x*, *u*_*m*_, *z* of system (5) are the voltages across the capacitors *C*_1_, *C*_2_, *C*_3_, respectively. As shown in Figure 6 the main component of the circuit is the memristive device, which is realized by common electronic components as presented in Figure 7. Indeed the circuit in Figure 7 emulates the memristive device (2) because there are not any commercial off-the-shelf memristors in the market yet. Therefore, the input and output of the memristive device are both voltages in this implementation. Here the input, output, and the internal state of the memristive device (2) are *u*_*m*_, *y*_*m*_, and the voltage across the capacitor *C*_4_, respectively. Hence, the memristive device is characterized by the following circuital equations:

(6) w˙m=1R10C4um,ym=(R14R11+R14100R12wm2−R14104R13wm4)um.

By using Kirchhoff's circuit laws, the equations of the circuit in Figure 6 are derived as follows:

(7) x˙=−1R1C1x−1R2C1um−110R3C1umz,u˙m=−1R4C2x+110R5C2xz+1R6C2ym−1R7C2Vb,z˙=−1R8C3z−110R9C3xum,w˙m=1R10C4um.

The power supplies are ±15 volts and the values of components are selected as *R*_1_ = *R*_3_ = 1.8 kΩ, *R*_2_ = 3.6 kΩ, *R*_4_ = 3 kΩ, *R*_5_ = *R*_9_ = 1.5 kΩ, *R*_6_ = 180 kΩ, *R*_7_ = 1.8 MΩ, *R* = *R*_8_ = *R*_10_ = *R*_11_ = *R*_14_ = 18 kΩ, *R*_12_ = 0.75 kΩ, *R*_13_ = 1.125 kΩ, *C*_1_ = *C*_2_ = *C*_3_ = *C*_4_ = 10 nF, and *V*_*b*_ = 0.1V_DC_.

**Figure 8:**
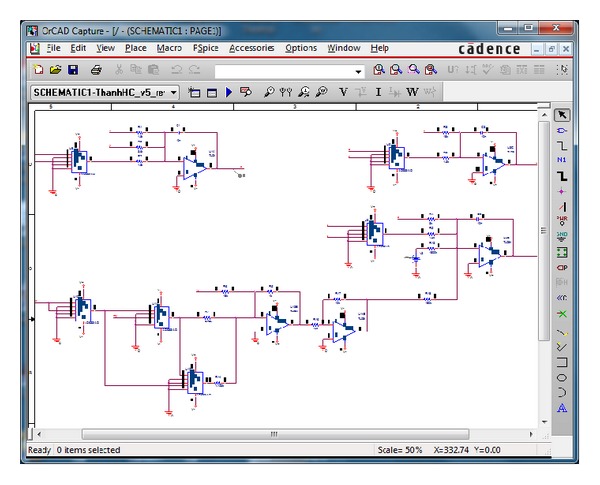
OrCAD schematic of the new hyperchaotic system without equilibrium (5).

**Figure 9:**
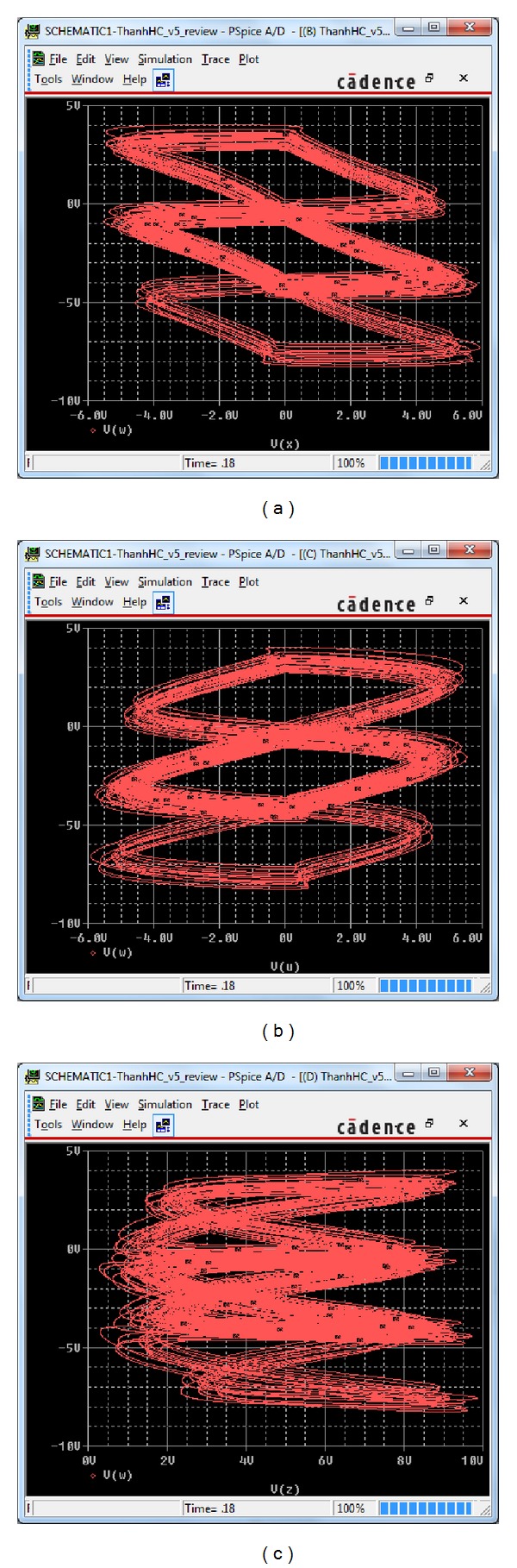
Hyperchaotic attractor of the designed electronic circuit obtained from OrCAD (a) in the *x* − *w*_*m*_ plane, (b) in the *u*_*m*_ − *w*_*m*_ plane, and (c) in the *z* − *w*_*m*_ plane.

The designed circuit is implemented in the electronic simulation package OrCAD (see Figure 8) and the obtained results are presented in Figure 9. It is easy to see a good agreement between the theoretical attractor (Figure 2) and the circuital one (Figure 9).

## 5. Conclusions

The existence of a memristor-based chaotic system without equilibrium has been studied in this paper. Although four-dimensional memristive systems often only generate chaos, the presence of a memristive device leads the proposed system to a hyperchaotic system with hidden attractors. The system has a rich dynamical behavior as confirmed by the examples of attractors reported and by the numerical Poincaré map presented. Because there is little knowledge about the special features of such system, future works will continue focusing on dynamical behaviours as well as the possibility of control and synchronization of such system.

Despite the fact that equations (2) do not correspond to a physical system, it is also true that memristive properties are today appearing in diverse devices and systems [38, 39]. Discovering meaningful physical system will be a future work. In this work, an analog circuit emulating the memristive device has been designed. However, alternative approaches for emulating this memristive device, that is, a microcontroller-based memristor emulator [40], can be applied to increase the robustness of the circuit.
